# Suction cup denture for restoration of maxillary edentulousness with hemilateral maxillary defects: a case report

**DOI:** 10.3389/fdmed.2025.1686431

**Published:** 2025-10-27

**Authors:** Kaijie Lin, Yanhui Wang, Jianxin Wang, Lei Ma

**Affiliations:** ^1^ Department of Prosthodontics, The Affiliated Hospital of Qingdao University, Qingdao, China; ^2^ School of Stomatology, Qingdao University, Qingdao, China; ^3^ Department of Oral Implantology, The Affiliated Hospital of Qingdao University, Qingdao, China

**Keywords:** maxillary defect, hemimaxillectomy, maxillofacial prosthesis, denture retention, suction cup denture, follow-up studies

## Abstract

This single case report describes the rehabilitation of a 71-year-old man with a hemimaxillary defect following resection of maxillary gingival carcinoma and reconstruction with a pedicled submental island flap. Conventional prosthetic rehabilitation had failed to provide adequate retention, and implant therapy was contraindicated due to prior radiotherapy. A suction cup denture was fabricated as a minimally invasive alternative to restore function. At six months, the denture demonstrated satisfactory retention, stability, and masticatory efficiency, with the patient reporting improved comfort, speech, and quality of life, and no persistent mucosal complications were observed. This case suggests that suction cup dentures may serve as a temporary, low-cost, and functionally effective option for hemimaxillectomy patients with specific indications, provided that wearing time is restricted and close follow-up is maintained to minimize risks.

## 1 Introduction

Rehabilitation after maxillectomy is complex, particularly when implant therapy is contraindicated due to radiotherapy, chemotherapy, or systemic comorbidities (1). In such situations, surgical reconstruction may be limited or delayed, and oral function must rely mainly on prosthodontic approaches. Prosthodontic rehabilitation therefore becomes the primary option. However, in hemimaxillectomy patients with flap transplantation, the presence of an edentulous maxilla, combined with insufficient teeth and bone support and the absence of usable tissue undercuts, often means that conventional obturators or dentures fail to provide adequate retention and stability (2, 3). Suction cup dentures have been reported as a minimally invasive, low-cost alternative in selected cases, offering temporary improvement in retention, function, and patient satisfaction when other options are not feasible. This report describes the application of a suction cup denture in a hemimaxillectomy patient and aims to provide clinical insights for similar complex restorative cases (4).

## 2 Case report

### 2.1 Detailed medical record information

A 71-year-old male patient presented with the chief complaint of loss of maxillary dentition for more than 1 year. One year prior, the patient underwent left maxillary extended resection and submental island flap-transfer repair for squamous cell carcinoma of the left maxillary gingiva. Postoperative pathology revealed well-differentiated (G1) squamous cell carcinoma of the left maxillary gingiva with bone invasion (pT4aN0M0). The patient subsequently received image-guided intensity-modulated radiotherapy (IG-IMRT) to a total dose of 54 Gy in 30 fractions (1.8 Gy per fraction). The patient visited our department for the repair of maxillary dentition, to restore mastication. It is noteworthy that the patient had remained edentulous for approximately one year since surgery, which significantly affected mastication and quality of life. His personal and family medical histories were unremarkable.

### 2.2 Clinical examination

#### 2.2.1 Extra-oral examination

**Figure 1:**
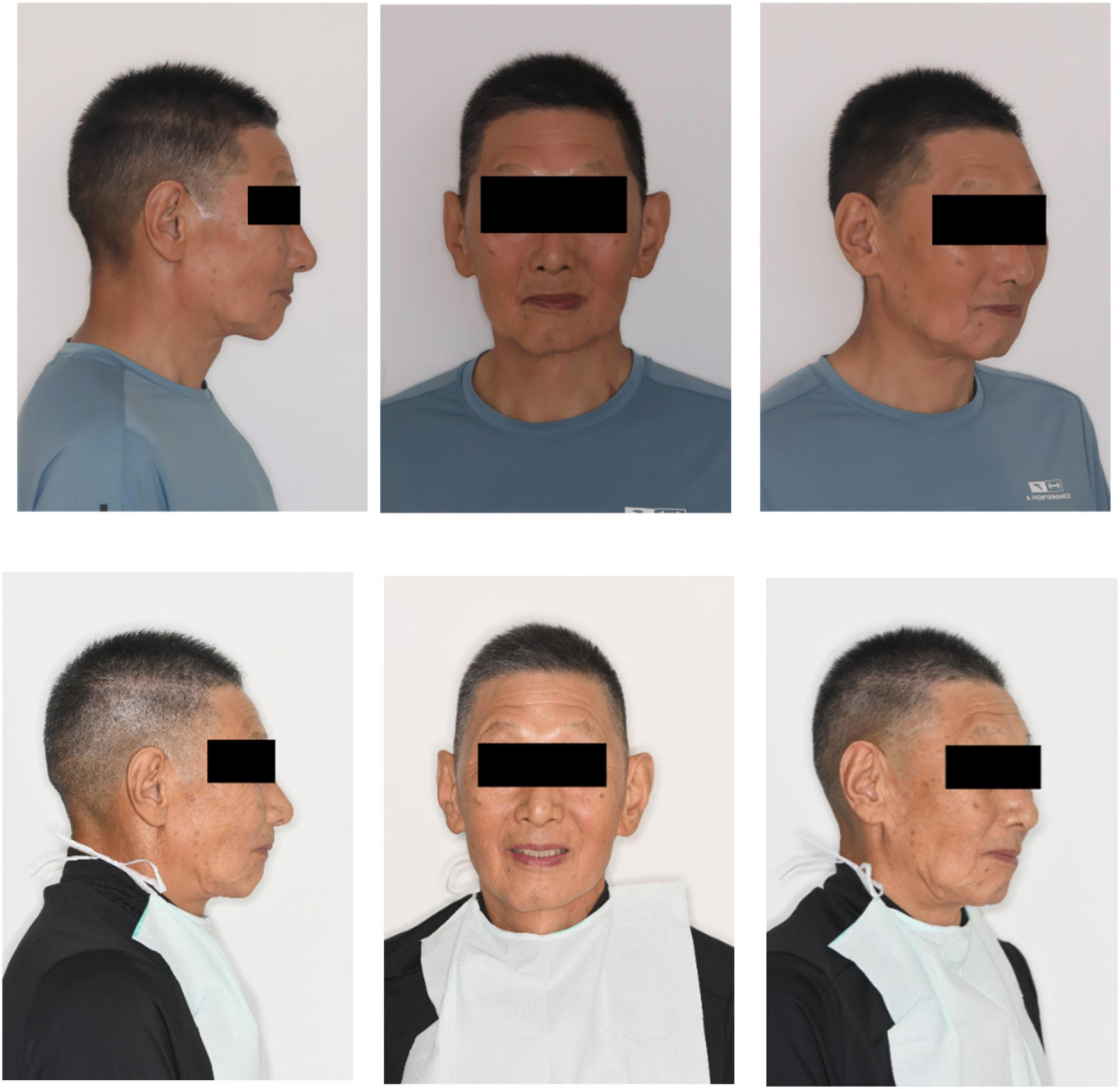
Preoperative (top) and postoperative (bottom) facial photographs of the patient.

The lower third of the face exhibited a slightly reduced height with mild drooping of the mouth angle. Asymmetry was noted between the left and right sides of the lower lip, and a surgical scar was visible on the midline of the lower lip and chin. No clicking or tenderness was detected in the temporomandibular joints bilaterally. The maximum mouth opening measured was approximately three finger-widths, and the pattern of mouth opening was normal (Figure 1-top).

#### 2.2.2 Intraoral examination

**Figure 2:**
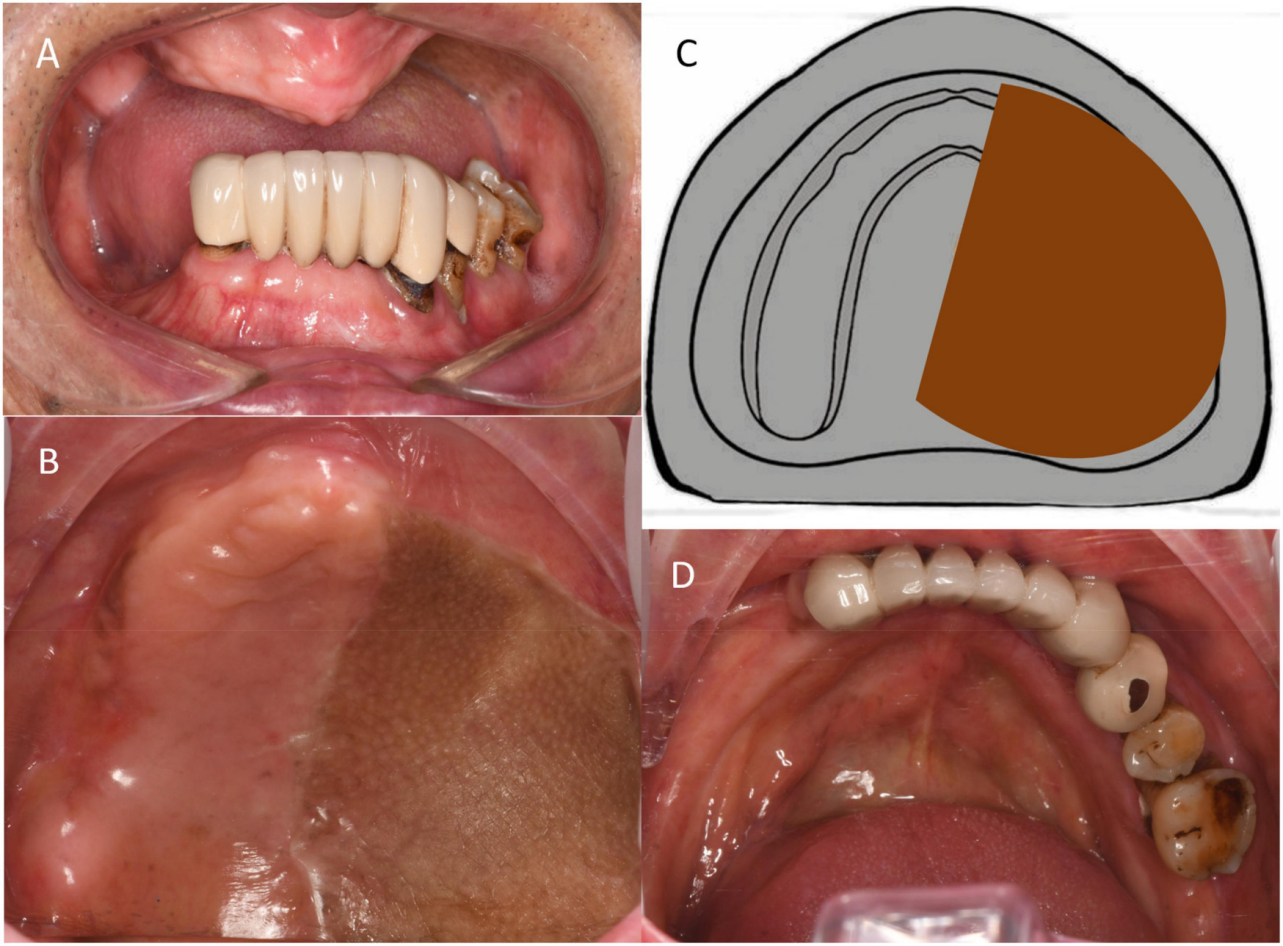
Preoperative intraoral photographs. **(A)** Frontal occlusal view. **(B)** Maxillary intraoral view. **(C)** Schematic representation of Aramany class-II maxillary defect. **(D)** Occlusal view of the mandible.

Maxillary edentulism (Figure 2A) and Aramany-II maxillary defects (Figures 2B,C) were observed in the left maxilla. The bone defects involved the hard palate, alveolar bone, gingiva, and mucosa. The remaining alveolar ridge on the right side of the maxilla was low and the molar area was approximately at the level of the maxillary basal bone (Figure 2B).

The mandibular dentition defect comprised edentulous right first premolar-to-second molar and left second molar areas and fixed bridge restoration in the left first premolar to the right canine areas. The edge of the prosthesis did not fit properly, loosening was of I–II degree, gingiva was slightly red and swollen, and left mandibular alveolar ridge was low and flat (Figure 2D).

The upper and lower alveolar ridges were not significantly convex or cusp-shaped, and the labio-buccal groove was unclear. The positional relationship between the maxillary and mandibular jaws was almost normal.

### 2.3 Imaging studies

(Figure 3).

**Figure 3:**
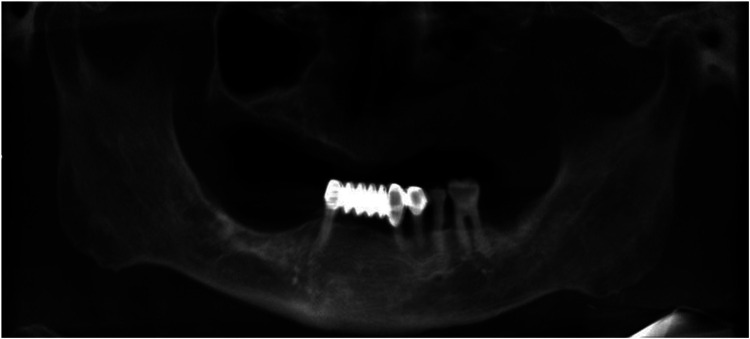
Initial cone-beam computed tomography image.

#### 2.3.1 Diagnosis

The patient was diagnosed with edentulous maxilla, maxillary defect of Aramany class II (5), and mandibular dentition defect.

### 2.4 Treatment plan options

#### 2.4.1 Scheme 1: suction cup denture

The elastic suction mechanism on the palate generates adhesion force, thereby enhancing the retention and stability of the denture. However, due to the significant adsorption pressure exerted by the suction cup on the local mucosa, its use may be limited to mealtimes or other necessary occasions. This approach demands a high level of patient compliance.

#### 2.4.2 Scheme 2: implant-supported dentures

Implant-supported dentures provide superior retention and masticatory function but are associated with higher costs and a longer treatment duration. Considering that the patient underwent postoperative radiotherapy following the doctor's instructions, there was an increased risk of complications, such as osteonecrosis following radiotherapy (6).

Considering the patient's preference for a restoration method with a shorter treatment duration and minimal invasiveness and based on the patient's intraoral condition and interdisciplinary communication, we decided to proceed with the suction cup denture restoration.

### 2.5 Treatment procedure

**Figure 4:**
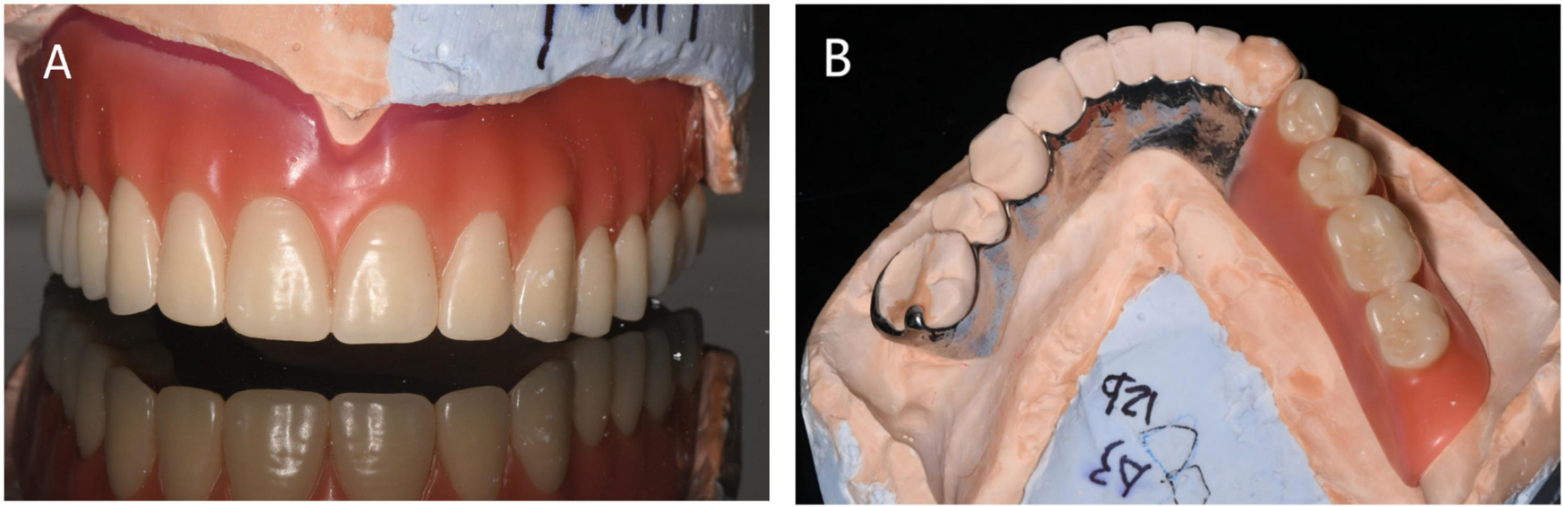
Rendering of the denture wax type. **(A)** Buccal view of the wax-shaped maxillary denture. **(B)** mandibular denture.

**Figure 5:**
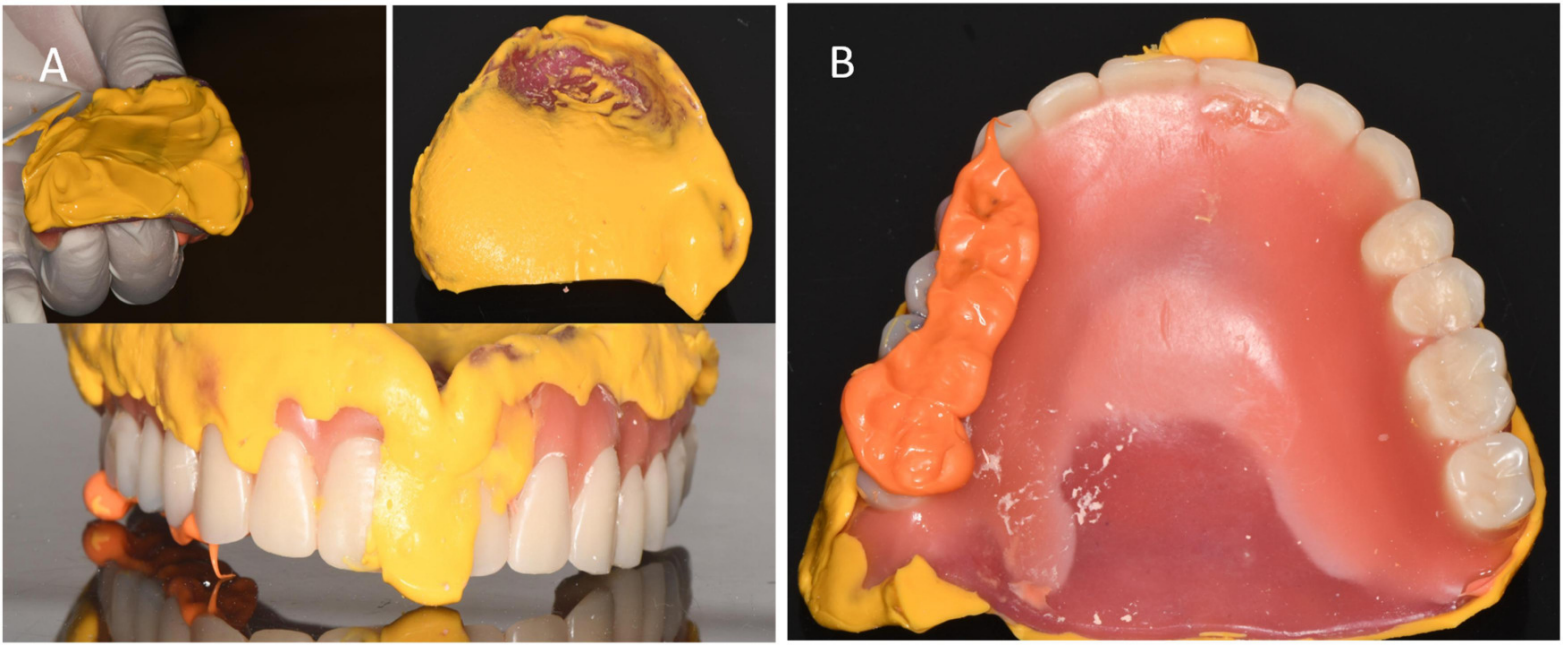
Recording of closed-mouth impression. **(A)** Closed impression recorded with silicone rubber. **(B)** Recorded occlusal relationship.

1. Preparation of primary impressions and individual trays: A stock tray suitable for the patient's dental arch was carefully selected. The alginate material was utilised to record the primary impression, followed by plaster pouring into a mould to produce the initial cast. The edge lines for individual trays were drawn meticulously on the plaster model, and a light-cured resin was employed to fabricate the individual trays.
2. Determination of final impressions and jaw relationships: Silicone rubber was used to record the final impression, and a working model was obtained through superanhydrite perfusion. Vertical and horizontal distances were accurately determined using the jaw gap and swallowing bite methods. Midline and oral commissure lines were established, and the occlusal relationship was recorded prior to denture fabrication.
3. Occlusal design: Considering that the defect cannot be forcibly corrected, the denture may be prone to twisting and swinging. In the occlusal design, the principles of complete denture construction were adhered to, ensuring balanced occlusion in the centric, protrusive, and lateral positions. This guarantees that the denture achieves a stable and balanced occlusal relationship.
4. Trial of the denture wax pattern: The denture wax pattern (Figure 4) was used to verify the appropriate extension of the denture base margins, tenderness of the tissue surface, accuracy of the occlusal relationship, and whether the centric, protrusive, and lateral balanced occlusions were achieved. The patient's facial profile and vertical dimension were also assessed. To enhance the fit of the denture, an individual tray was fabricated using the maxillary wax pattern to record a closed functional impression (Figure 5), and the occlusal relationship was confirmed. A suction cup retainer ring was designed at the midpoint of the maxillary first molar, and the silicone rubber piece was fixed in the groove of the suction cup on the retainer ring.
5. Initial denture placement and professional guidance: The position and retention of the denture were evaluated, checking for any lifting or tenderness, whether the base edge is excessively long, and if the frenulum buffer is adequate. Additionally, the patient's facial profile was assessed, ensuring that the vertical dimension was appropriate, jaw relationship was correct, and that the denture achieved balanced occlusion (Figures 1-bottom, 6, and 7). The use of dentures should be restricted due to the adhesion and negative pressure exerted on the palatal mucosa by the suction cup. The patient was advised to wear them only while eating and chewing, using the right side for these activities. Furthermore, close attention was paid to palatal mucosal conditions.

**Figure 6:**
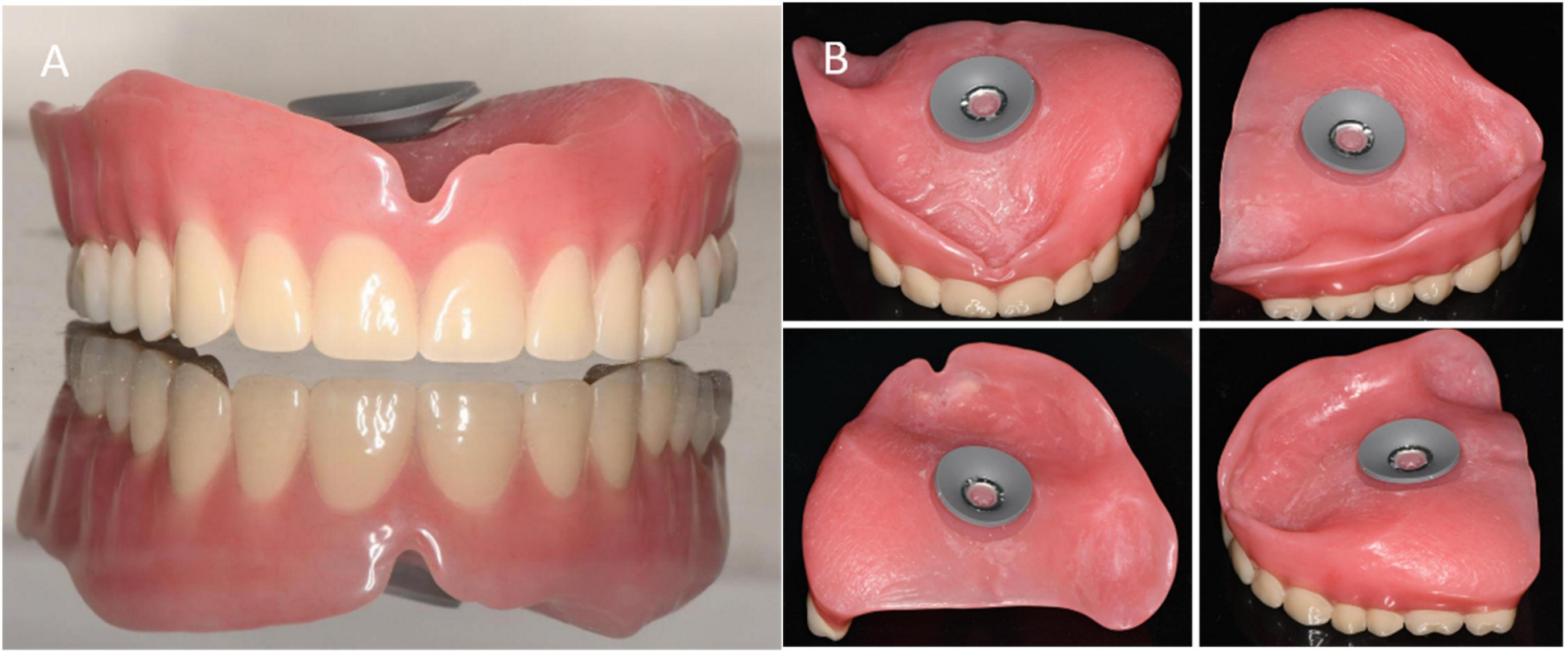
Suction cup denture. **(A)** The front view of the maxillary denture. **(B)** The tissue surface of the maxillary denture.

**Figure 7:**
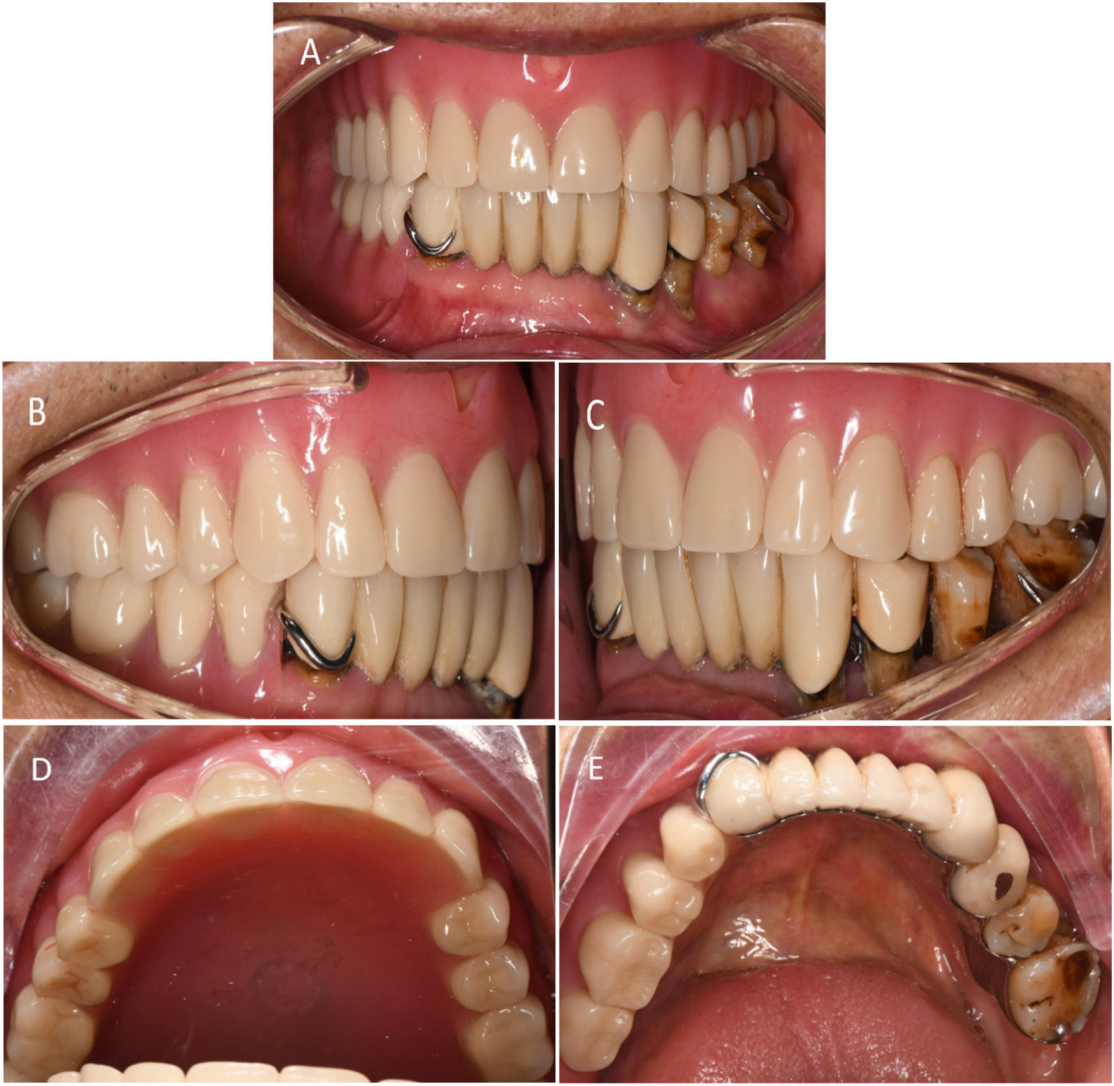
Postoperative photographs. **(A)** Buccal view of denture. **(B)** Right-side occlusion (non-defect side). **(C)** Left-side occlusion (defect side). **(D)** Occlusal view of the maxillary denture. **(E)** Occlusal view of the mandibular denture.

### 2.6 Outcomes

**Figure 8:**
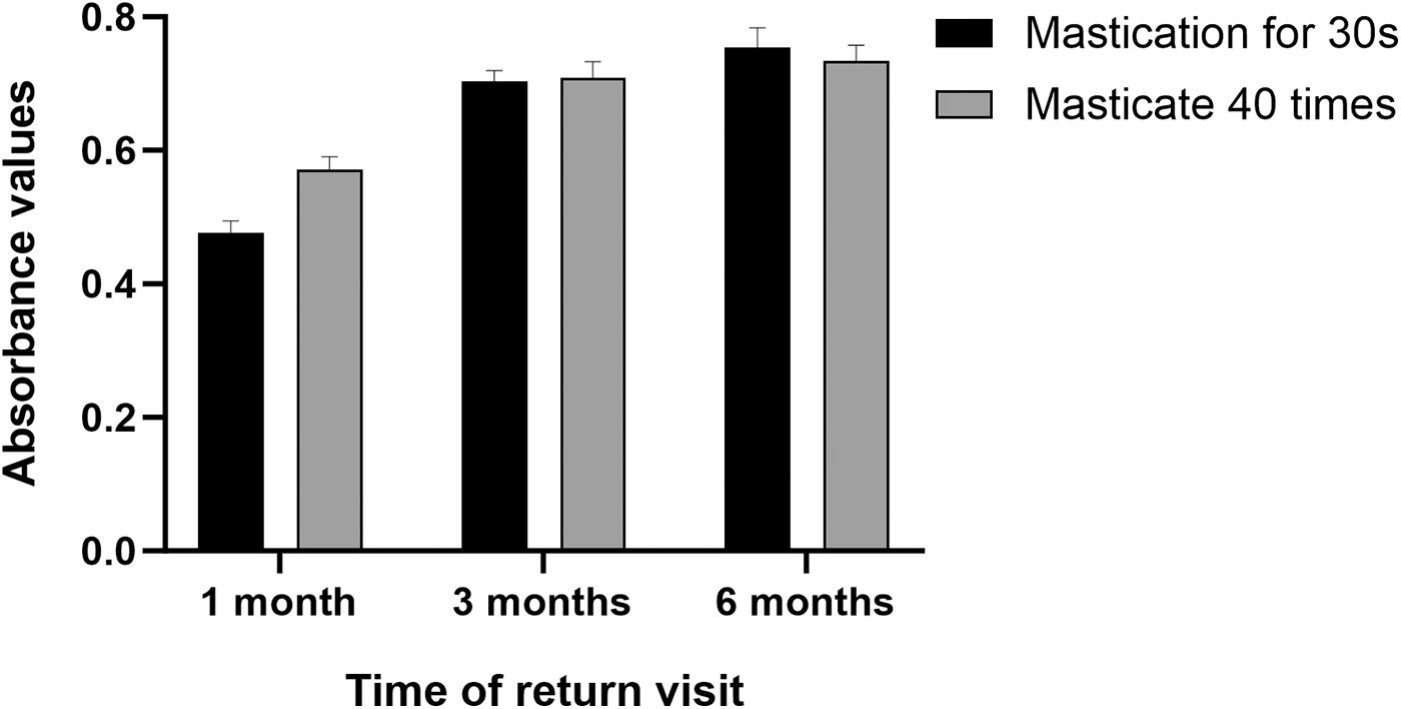
Absorbance values following mastication using two methods, with timing of 30 s and 40 silent counts for 5 g of peanuts.

**Figure 9:**
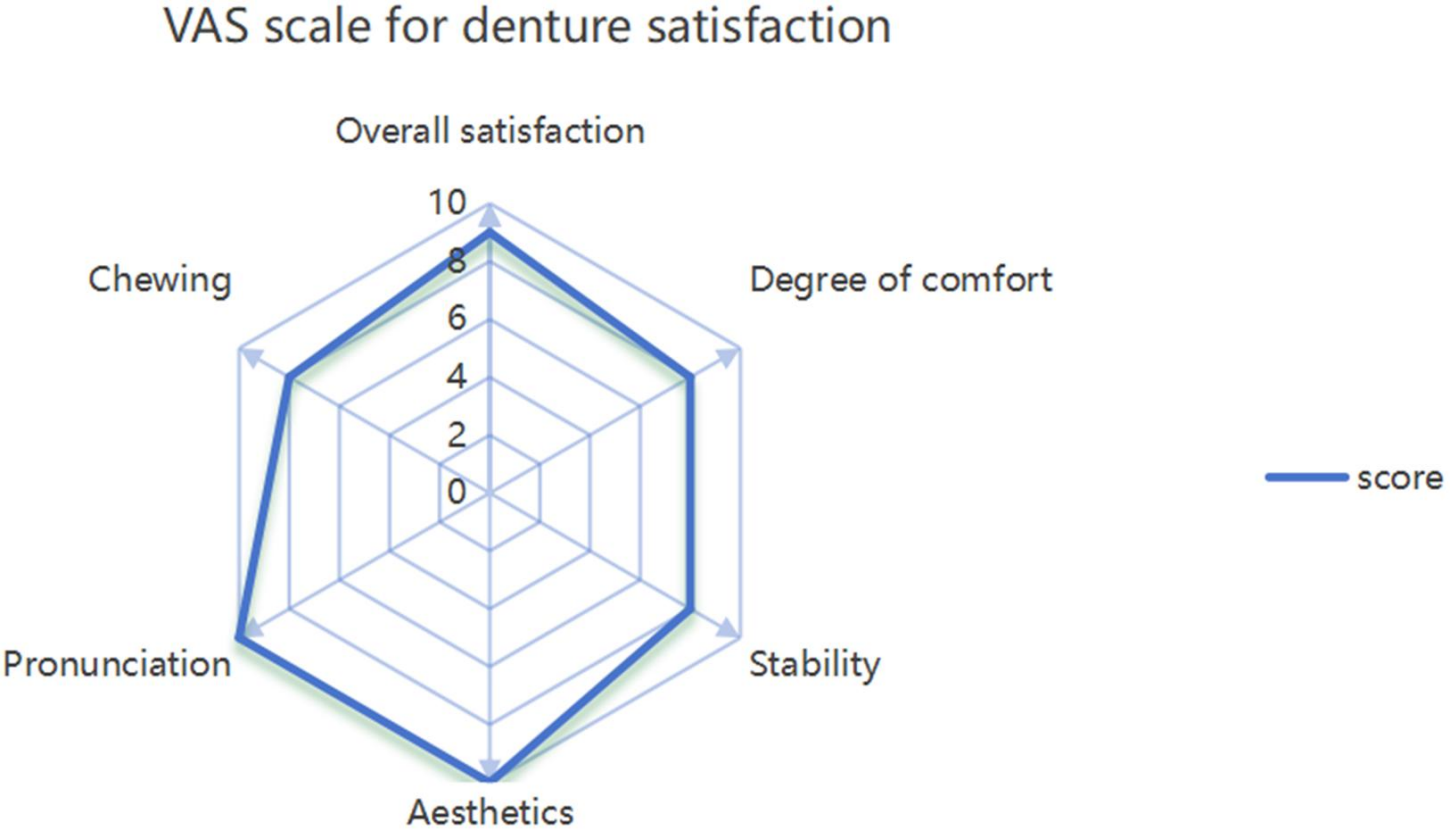
Visual analogue scale score table to evaluate patient satisfaction with denture.

Using the peanut-absorbance test (both 30-s timed and 40-stroke counted protocols), efficiency increased from month 1 to month 3 and was maintained at month 6, remaining higher than at month 1 (Figure 8). Functionally, the patient could eat steamed bread, noodles, raw apple, peanuts, and tender meat without pain. The satisfaction scale was administered at months 1, 3, and 6. To summarize the single-patient trajectory without over-interpreting minor fluctuations, we report the mean across the three visits, which indicated acceptable-to-good satisfaction (Figure 9). The Modified Oral Mucosal Score (0–3 per domain; total 0–18) remained low: total scores were 0 at baseline, 1 at month 1 (grade-1 indentation), 2 at month 3 (grade-1 indentation plus grade-1 swelling), and 1 at month 6 (residual grade-1 indentation only); tenderness, erythema, ulceration, and white lesions were 0 at all visits (Table 1).

**Table 1:** Palatal mucosa condition scoring using the Modified Oral Mucosal Score (OMS, 0–3 scale).

Time point	Tenderness	Indentation	Erythema	Swelling	Ulceration	White lesions	Total score (0–18)
Baseline (0 month)	0	0	0	0	0	0	0
1 month	0	1 (mild)	0	0	0	0	1
3 months	0	1 (mild)	0	1 (mild)	0	0	2
6 months	0	1 (mild)	0	0	0	0	1

0 = none; 1 = mild; 2 = moderate; 3 = severe.

#### 2.6.1 Masticatory efficiency assessment

A spectrophotometer was employed to evaluate masticatory efficiency using red peanuts as the test material. The skin and pedicle were removed from the peanuts, and a 5 g sample was prepared. The participant chewed the peanuts according to two methods: either timed for 30 s or counted tacitly for 40 chews, after which the chewed material was expectorated into a container. Any residual material in the mouth or dentures was rinsed and collected in the same container. The collected samples were diluted to 1,000 ml with distilled water, thoroughly mixed for 1 min, and allowed to settle for 2 min. Aliquot of the suspension from the midpoint (approximately 60 ml) was aspirated and analysed using a spectrophotometer. The light source of the instrument emitted monochromatic light at a wavelength of 590 nm. In this study, the absorbance values reported represent the mean of three repeated measurements for the same patient at each time point. Higher absorbance values indicated greater particle dispersion, whereas lower values suggested less efficient mastication. This method was used to assess the masticatory efficiency of dentures (7) (Figure 8).

#### 2.6.2 Clinical examination of the mucosa

**Figure 10:**
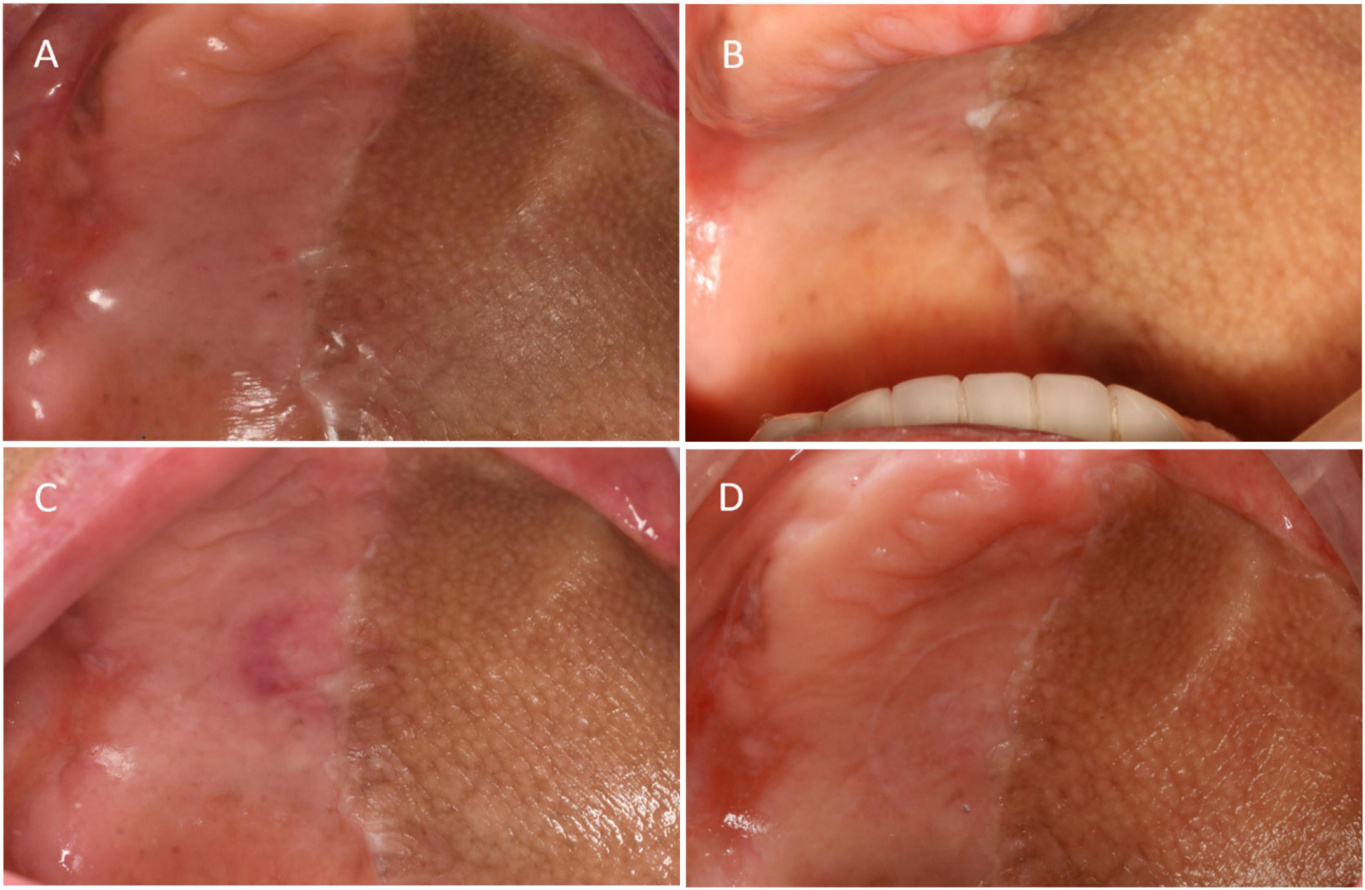
Palatal mucosal condition. **(A)** Palatal mucosa before wearing the denture. **(B)** Palatal mucosa after wearing the denture for 1 month. **(C)** Palatal mucosa after 3 months of wearing the denture. **(D)** Palatal mucosa after 6 months of wearing the denture.

This case specifically focused on the palatal mucosa at the suction cup site, which was assessed using the Modified Oral Mucosal Score (OMS, 0–3 scale; 0 = none, 1 = mild, 2 = moderate, 3 = severe) at baseline (A) and at 1 month (Figure 10B), 3 months (Figure 10C), and 6 months (Figure 10D) after denture insertion. OMS scores are summarized in Table 1, showing consistently low levels without persistent mucosal complications (8, 9) (Table 1).

#### 2.6.3 Satisfaction score

Patient satisfaction with the denture was evaluated, and the visual analogue scale of denture satisfaction was used to score the stability of the denture, mastication, comfort, pronunciation, aesthetics, and overall satisfaction, with a total score of 60 (Figure 9).

## 3 Discussion

Following oncologic resection, maxillary defects are frequently extensive and anatomically complex, which in turn undermines oral function—mastication, deglutition, and speech (10). In these cases, denture restoration is critical. The extent of the maxillary defect, volume of remaining bone structure, number of remaining teeth, and efficacy of radiotherapy collectively influence the selection of the repair method (11). Maxillary edentulism with hemimaxillary defect represents one of the several types of maxillofacial defects. Treatment primarily involves surgical reconstruction or prosthetic restoration (2). Compared to surgical reconstruction, prostheses play an indispensable role in the rehabilitation of maxillary defects because of their minimal risk, cost-effectiveness, and broad applicability. However, conventional prostheses rely on the residual teeth or undercuts of soft and hard tissues to achieve retention (2). In cases where the maxilla is edentulous and the defect is repaired using a flap, although oronasal communication is prevented, the undercut of the defect is also eliminated. Consequently, traditional prostheses do not utilise natural teeth, or tissue undercuts to achieve adequate retention. Implant restoration is considered an ideal rehabilitation approach. However, most patients with malignant tumours undergo high-dose radiotherapy following surgical treatment. Studies have indicated that the failure rate of dental implants in patients with head and neck malignancies who receive postoperative radiotherapy is relatively high (approximately 11%). This is primarily attributed to radiation-induced osteonecrosis (12). The risk of osteoradionecrosis (ORN) remains elevated in irradiated bone regardless of the time elapsed after radiotherapy. Although delaying implant placement beyond 12 months may improve survival, long-term studies still show higher implant failure and ORN rates compared with non-irradiated patients (13, 14). Thus, even one year after 54 Gy IMRT, as in this case, implant therapy carries persistent risk, supporting the choice of prosthodontic rehabilitation over implants.

After total hemimaxillary resection and flap repair, oral anatomical structures on the defect side—such as the vestibular sulcus—are lost and the marginal mucosa is highly mobile, making a reliable border seal difficult to achieve. Consequently, conventional complete-denture retention based on salivary adhesion and border-seal negative pressure cannot be established (15). In this anatomic context, obturators are also suboptimal: the bulk required for obturation and the absence of a stable sulcus compromise retention and increase rotational tendencies (16). Therefore, a suction cup denture was selected in this case to enhance retention and stability. The suction cup structure is generally placed at the midpoint of the connection line of the maxillary first molars, and atmospheric pressure is used to provide sufficient retention force for the denture when it is fully positioned (17). Although this device may resemble the historical rigid acrylic suction chambers that were abandoned because of mucosal injury and palatal fistulas (18), the present approach differs in material (elastic rather than rigid acrylic), size (smaller footprint with rounded margins), and protocol (strictly limited to mealtimes with close follow-up). In this patient, the retention of the denture was significantly enhanced following the implementation of suction cups. The denture was secured in place during rest, speech, eating, and mastication, thereby achieving the expected therapeutic outcomes.

In this case, the left side of the patient's maxilla was covered solely by the mucosa without underlying bone support, resulting in unilateral occlusal support of the denture. Additionally, the maxilla was completely edentulous, whereas the mandible retained its natural teeth, due to which achieving balanced occlusion was challenging. Prior to occlusal examination and adjustment, the overextension of the denture margins and any early contact areas on the tissue surface were thoroughly adjusted to prevent instinctive avoidance of dentures due to abnormal local movements. Examination and adjustment of the jaw relationships during centric, protrusive, and lateral excursions are essential, with a focus on establishing balanced occlusion (19). Some studies (20, 21) have indicated that in cases where the jaw–arch relationship is suboptimal, the restoration outcome of a lingual concentrated jaw arrangement surpasses that of other jaw types, with significant improvements in masticatory efficiency and patient comfort. In the described scenario, the elongation of the remaining natural teeth in the lower jaw and abnormality of the occlusal curve made establishing an effective lingual concentrated jaw challenging. Nevertheless, for edentulous patients exhibiting similar alveolar ridge damage, the implementation of a lingual concentrated jaw may be considered a viable option to enhance denture stability.

Restoration of masticatory function was of substantial significance in this case. The masticatory function was assessed by inquiring regarding the patient's dietary structure, and masticatory efficiency was evaluated using the peanut-chewing absorbance method during follow-up visits. This method is characterised by its simplicity, rapidity, ease of material acquisition, and excellent repeatability. Using both time-controlled (30 s) and stroke-controlled (40 strokes) chewing allowed complementary evaluation: the timed method reflects natural mastication, while the stroke method reduces variability and improves comparability. Consistent trends across both methods enhanced the reliability of masticatory efficiency assessment (22). The absorbance values reached high levels by the third month. In the sixth month, no significant change was observed compared to that in the third month, which was higher than the value observed in the first month. These findings indicate that the patient's masticatory function can achieve a relatively stable state after denture adjustment.

The suction force exerted by the suction cup places a greater mechanical load on the mucosa than on other tissues, and prolonged exposure to excessive forces can readily result in localised mucosal lesions. Consequently, the condition of the mucosa at the suction site was the primary focus of the present study. Several case reports have highlighted (18) that the use of suction cups in complete maxillary dentures frequently induces localised mucosal abnormalities, such as erythema, oedema, leucoplakia, and even palatal fistulas, which may lead to oronasal communication and significantly impair the quality of life of patients. However, studies involving patients with palatal fistulas have indicated that suction cup dentures are often worn ontinuously for more than a decade (23), both day and night, with removal occurring solely for cleaning purposes. The authors believe that the key cause of palatal fistulas is prolonged unscientific wearing. Considering also that patients with advanced maxillary gingival squamous cell carcinoma, such as stage IVB in this case, generally have a limited 5-year survival rate of about 58% (24), many long-latency mucosal complications may not have sufficient time to develop. A 2-year follow-up study of sucker dentures (4) revealed mild erythema of the local mucosa, with no signs of bleeding or erosion. Appropriate and scientifically guided use does not lead to the development of local mucosal lesions. One patient was monitored for 6 months, during which localised indentation and erythema were observed but without swelling or tenderness.

This is a single-patient report with short follow-up (6 months), Mucosal safety observed here is contingent on device design and strict, limited wear; risks may rise with prolonged or continuous use. Generalizability across defect patterns, radiation doses, and reconstructions is therefore restricted. The authors suggested extending the observation period to further evaluate the long-term effects. In addition, patients should be consistently reminded to restrict their wearing time, use dentures only in necessary situations, adhere to appropriate eating practices while wearing them, and maintain rigorous cleaning protocols.

## 4 Conclusion

In carefully selected hemimaxillectomy patients for whom implants are contraindicated, a limited-wear suction cup denture can provide short-term functional rehabilitation. In this single case, masticatory efficiency improved and stabilized, while palatal mucosal scores remained low under a strict wear protocol and regular review. This approach should be regarded as a temporary, minimally invasive bridge to function, not a definitive solution. Close follow-up and adherence to restricted wearing time are essential to minimize mucosal complications. Therefore Suction dentures may be an option in carefully selected cases but require strict follow-up to prevent complications.

## Data Availability

The raw data supporting the conclusions of this article will be made available by the authors, without undue reservation.

## References

[1] Fan S Diaz L Sáenz-Ravello G Valmaseda-Castellon E Al-Nawas B Schiegnitz E. Comprehensive update on implants in patients with head and neck cancer (2021–2024): systematic review and meta-analysis of the impact of radiotherapy and chemotherapy on implant survival. Clin Oral Implants Res. (2025) 36(9):1035–52. 10.1111/clr.14450 40530953 PMC12423586

[2] Barraclough O Patel J Milne S Ho MW Ali Z. Pathways for the rehabilitation of resection defects in the maxilla. Br Dent J. (2022) 232(11):783–9. 10.1038/s41415-022-4342-3 35689055

[3] Butterworth C. The prosthodontic management of the maxillectomy patient. Br Dent J. (2022) 233(9):744–8. 10.1038/s41415-022-5106-9 36369555

[4] Li XYXL Liu WF Liu X. Clinical observation of elastic suction discs in complete maxillary denture rehabilitation. J Dent Prev Treat (Guangdong). (2011) 19(2):94–7. cnki:sun:gdyb.0.2011-02-011

[5] Alqarni H Alfaifi M Ahmed WM Almutairi R Kattadiyil MT. Classification of maxillectomy in edentulous arch defects, algorithm, concept, and proposal classifications: a review. Clin Exp Dent Res. (2023) 9(1):45–54. 10.1002/cre2.708 36600487 PMC9932229

[6] Lajolo C Rupe C Gioco G Troiano G Patini R Petruzzi M Osteoradionecrosis of the jaws due to teeth extractions during and after radiotherapy: a systematic review. Cancers (Basel). (2021) 13(22):5798. 10.3390/cancers13225798 34830954 PMC8616343

[7] Dula LJ Shala KS Stubljar D Starc A Kosumi S. Comparison of increase in masticatory efficiency between removable partial dentures retained with clasps and retained with attachments. Clin Exp Dent Res. (2025) 11(2):e70130. 10.1002/cre2.70130 40260841 PMC12012738

[8] Khanagar S Naganandini S Rajanna V Naik S Rao R Madhuniranjanswamy MS. Oral hygiene Status of institutionalised dependent elderly in India - a cross-sectional survey. Can Geriatr J. (2015) 18(2):51–6. 10.5770/cgj.18.147 26180560 PMC4487736

[9] Schubert MM Williams BE Lloid ME Donaldson G Chapko MK. Clinical assessment scale for the rating of oral mucosal changes associated with bone marrow transplantation. Development of an oral mucositis index. Cancer. (1992) 69(10):2469–77. 10.1002/1097-0142(19920515)69:10<2469::AID-CNCR2820691015>3.0.CO;2-W 1568168

[10] Farghal AE. Fabrication of a definitive obturator for a patient with a maxillary defect: a case report. Cureus. (2023) 15(12):e50578. 10.7759/cureus.50578 38222126 PMC10788096

[11] Aladashi OQS Shindy MI Amin AA Noaman SA Alqutaibi AY Behery MG Corrigendum to ‘effect of submental flap reconstruction versus obturator rehabilitation after maxillectomy on quality of life: a randomized clinical trial’ [Int J Oral Maxillofac Surg, 50 (9) (2021), 1156–1160]. Int J Oral Maxillofac Surg. (2023) 52(1):e1. 10.1016/j.ijom.2022.10.006 36266167

[12] Jeong YJ Dunn M Manzie T Howes D Wykes J Palme CE Jaw in a day surgery: early experience with 19 patients at an Australian tertiary referral center. ANZ J Surg. (2024) 94(9):1531–8. 10.1111/ans.19203 39158220

[13] Singh A Kitpanit S Neal B Yorke E White C Yom SK Osteoradionecrosis of the jaw following proton radiation therapy for patients with head and neck cancer. JAMA Otolaryngol Head Neck Surg. (2023) 149(2):151–9. 10.1001/jamaoto.2022.4165 36547968 PMC9912132

[14] Toneatti DJ Graf RR Burkhard JP Schaller B. Survival of dental implants and occurrence of osteoradionecrosis in irradiated head and neck cancer patients: a systematic review and meta-analysis. Clin Oral Investig. (2021) 25(10):5579–93. 10.1007/s00784-021-04065-6 34401944 PMC8443505

[15] Thalji G McGraw K Cooper LF. Maxillary complete denture outcomes: a systematic review of patient-based outcomes. Int J Oral Maxillofac Implants. (2016) 31(Suppl):s169–81. 10.11607/jomi.16suppl.g5.1 27228248

[16] Singh M Bhushan A Kumar N Chand S. Obturator prosthesis for hemimaxillectomy patients. Natl J Maxillofac Surg. (2013) 4(1):117–20. 10.4103/0975-5950.117814 24163568 PMC3800374

[17] Zhao W. Clinical observation of elastic mini suction disc in restoring maxillary edentulism: a report of 42 cases. Chin Foreign Med Res. (2013) 11(8):127. 10.14033/j.cnki.cfmr.2013.08.122

[18] Rao Y Yadav P Singh J Patel D Aggarwal A. Surgical and prosthetic management of suction cup induced palatal perforation: case report. J Clin Diagn Res. (2013) 7(9):2086–7. 10.7860/JCDR/2013/6300.3413 24179952 PMC3809691

[19] Koper A. The maxillary complete denture opposing natural teeth: problems and some solutions. J Prosthet Dent. (1987) 57(6):704–7. 10.1016/0022-3913(87)90367-2 3295202

[20] Wang Z Su Y Wang J Liu Y Xing W. Occlusal parameters and wear of artificial teeth in complete dentures with lingualized versus bilateral balanced occlusion: a randomized clinical trial. BMC Oral Health. (2024) 24(1):1405. 10.1186/s12903-024-05144-2 39563363 PMC11575096

[21] Kawai Y Ikeguchi N Suzuki A Kuwashima A Sakamoto R Matsumaru Y A double blind randomized clinical trial comparing lingualized and fully bilateral balanced posterior occlusion for conventional complete dentures. J Prosthodont Res. (2017) 61(2):113–22. 10.1016/j.jpor.2016.07.003 27474364

[22] Gonçalves T Schimmel M van der Bilt A Chen J van der Glas HW Kohyama K Consensus on the terminologies and methodologies for masticatory assessment. J Oral Rehabil. (2021) 48(6):745–61. 10.1111/joor.13161 33638156 PMC8252777

[23] Vaaka P Donga SK Ganapathi AK Devi PB Kaluvakolanu S Mohammad Z. Suction cup induced palatal fistula: surgical closure by palatal rotational flap. Ann Med Health Sci Res. (2016) 6(2):129–32. 10.4103/2141-9248.181842 27213097 PMC4866366

[24] Kang CJ Tsai CY Lee LY Lin CY Yang LY Cheng NM Prognostic stratification of patients with AJCC 2018 pStage IVB oral cavity cancer: should pT4b and pN3 disease be reclassified? Oral Oncol. (2021) 119:105371. 10.1016/j.oraloncology.2021.105371 34174527

